# Persistence of accuracy of genomic estimated breeding values over generations in layer chickens

**DOI:** 10.1186/1297-9686-43-23

**Published:** 2011-06-21

**Authors:** Anna Wolc, Jesus Arango, Petek Settar, Janet E Fulton, Neil P O'Sullivan, Rudolf Preisinger, David Habier, Rohan Fernando, Dorian J Garrick, Jack CM Dekkers

**Affiliations:** 1 Department of Genetics and Animal Breeding, Poznan University of Life Sciences, Wolynska st. 33, 60-637 Poznan, Poland; 2 Department of Animal Science and Center for Integrated Animal Genomics, Iowa State University, Ames, IA 50011-3150, USA; 3 Hy-Line International, Dallas Center, IA 50063, USA; 4 Lohmann Tierzucht GmbH, 27472 Cuxhaven, Germany

## Abstract

**Background:**

The predictive ability of genomic estimated breeding values (GEBV) originates both from associations between high-density markers and QTL (Quantitative Trait Loci) and from pedigree information. Thus, GEBV are expected to provide more persistent accuracy over successive generations than breeding values estimated using pedigree-based methods. The objective of this study was to evaluate the accuracy of GEBV in a closed population of layer chickens and to quantify their persistence over five successive generations using marker or pedigree information.

**Methods:**

The training data consisted of 16 traits and 777 genotyped animals from two generations of a brown-egg layer breeding line, 295 of which had individual phenotype records, while others had phenotypes on 2,738 non-genotyped relatives, or similar data accumulated over up to five generations. Validation data included phenotyped and genotyped birds from five subsequent generations (on average 306 birds/generation). Birds were genotyped for 23,356 segregating SNP. Animal models using genomic or pedigree relationship matrices and Bayesian model averaging methods were used for training analyses. Accuracy was evaluated as the correlation between EBV and phenotype in validation divided by the square root of trait heritability.

**Results:**

Pedigree relationships in outbred populations are reduced by 50% at each meiosis, therefore accuracy is expected to decrease by the square root of 0.5 every generation, as observed for pedigree-based EBV (Estimated Breeding Values). In contrast the GEBV accuracy was more persistent, although the drop in accuracy was substantial in the first generation. Traits that were considered to be influenced by fewer QTL and to have a higher heritability maintained a higher GEBV accuracy over generations. In conclusion, GEBV capture information beyond pedigree relationships, but retraining every generation is recommended for genomic selection in closed breeding populations.

## Background

Genomic selection is based on the estimation of breeding values using high-density marker data [1] and provides opportunities to enhance genetic improvement programs [2,3]. The resulting marker-based or genomic estimated breeding values (GEBV) exploit associations between markers and QTL (Quantitative Trait Loci) through linkage disequilibrium (LD) and linkage, along with the capture of pedigree relationships between animals [4]. In contrast, estimated breeding values (EBV) obtained with the pedigree-based BLUP animal model rely completely on pedigree-based genetic relationships. The accuracy of GEBV is expected to be more persistent across generations than that of pedigree-based EBV because marker-based relationships resulting from LD and linkage are expected to erode during the successive meioses at a slower rate than genetic relationships. The initial hopes that after a single training analysis, genomic selection would permit to completely abandon progeny testing and phenotyping for traits that are expensive or difficult to measure [1] appear unrealistic. Several simulation studies have been carried out to determine which factors affect the persistency of the accuracy of GEBV over generations. Sonesson and Meuwissen [5] observed a rapid decline in the accuracy of GEBV in the generation immediately following the discontinuation of phenotyping. The decline in accuracy over generations was greater at low marker densities. Solberg et al. [6] also reported that higher marker densities help maintain accuracy over generations. These results are in agreement with those of Habier et al. [4] who showed that 1,000 to 2,000 markers are sufficient to capture most pedigree relationships and that accuracy due to LD, which requires higher densities, is more persistent. Muir [7] showed that the accuracy of GEBV declines much more rapidly over generations with selection on GEBV compared to random selection. However, Sonesson and Meuwissen [5] showed that the effect of selection on accuracy was small if retraining was done every generation. Habier et al. [4] compared the decline in accuracy over generations for different methods of breeding value estimation and found that the Bayesian model averaging method Bayes-B of [1] outperformed fixed regression least squares and a model equivalent to genomic BLUP, i.e., the BLUP animal model using marker-based relationships. In addition, the persistency of accuracy was greater for all marker-based methods than for EBV obtained from standard pedigree-based BLUP analyses.

Simulation studies provide insight into some of the mechanisms that influence the accuracy of EBV but they rely on assumptions for data generation, the validity of which is unknown in real data, such as the number of QTL, the distribution of their effects, and the population history that contributed to the current LD structure. Except for Habier et al. [8], who showed the decay in accuracy with decreasing pedigree relationship, to our knowledge, no studies on the dynamics of changes in accuracy of GEBV over generations in real data are available in the literature. Thus, the objective of this study was to evaluate the accuracy of EBV and to quantify the persistence of accuracy over five generations using marker versus pedigree information in a commercial breeding line of layer chickens.

## Methods

### Data

The data on 16 economically important traits measured at early (e) or later (l) ages, along with marker genotype data, were collected for a brown-egg layer line: age of sexual maturity (eSM), body weight at late age (lBW), shell colour for first three eggs (eC3), at early (eCO) and late age (lCO), egg weight for first three eggs (eE3), at early (eEW) and late age (lEW), egg production at early (ePD) and late age (lPD), puncture score at early (ePS) and late age (lPS), albumen height at early (eAH) and late age (lAH), yolk weight at early (eYW) and late age (lYW). Early measurements were taken at 26-28 weeks of age. Late measurements were taken at 42-46 weeks of age on birds not culled after early measurements. Early and late egg quality measurements were record averages on three to five eggs. Observations deviating from the within-hatch generation mean by more than three standard deviations were excluded as outliers. All data used in this study were obtained from the routine breeding program data collection of Hy-Line Int. Birds were genotyped for 23,356 segregating SNP obtained from a custom high-density Illumina SNP panel (minor allele frequency > 0.025; maximum proportion of missing genotypes < 0.05; maximum mismatch rate between parent-offspring pairs < 0.05). No editing was done based on GC score. Genotyped birds within a generation included all males and females used for breeding (approximately 60 male and 310 female parents per generation) and some additional progeny. Data cumulated over generations are described in Table 1. The population was under selection for the 16 traits, using EBV from pedigree-based BLUP (PBLUP) up to generation 3 and on a combination of GEBV and phenotype afterwards.

**Table 1:** Numbers of individuals with phenotypic and marker genotype data available for training and validation per generation

	Training data*			
		
Generation	Cumulated number genotyped	Cumulated number genotyped with own record	Cumulated number of progeny with genotyped parents Early/Late	Number genotyped and with own record in validation data
0	365	0	0	-
1	777	295	2,443/342	322
2	1,215	618	4,892/804	295
3	1,628	913	7,562/1,287	357
4	2,108	1,273	9,319/1,686	274
5	2,708	1,563	11,486/2,455	262

*Training data included only phenotypes prior to a given generation; validation data consisted of hens from that generation with genotypes and own phenotypes; these hens contributed to training data in subsequent generations; the intention was to measure all traits for the genotyped birds, therefore they had very few missing values, at most 23 missing phenotypes for a give trait for 1,563 genotyped hens.

### Statistical analysis

In order to evaluate the accuracy of EBV (accuracy in progeny), training was done on all data accumulated up to a given generation and validation was on genotyped progeny with own phenotypes. To analyze the persistence of EBV accuracy (accuracy in future generations), training was done on data accumulated through generation 1 and validation used genotypes and phenotypes of progeny from each subsequent generation. The following methods were used to compute EBV:

1) PBLUP - pedigree-based animal model with REML estimates of variance components. Pedigree starting at generation -1 was used.

2) GBLUP - reduced animal model using the genomic relationship matrix estimated by the method in [9], with allele frequencies based on the full data set. This model allows for the inclusion of phenotypes of non-genotyped progeny of genotyped parents. See [10] for details.

3) BayesA - a Bayesian model averaging method described by [1].

4) BayesCπ - a Bayesian model averaging method similar to the Bayes-B method in [1] but including an estimation of the proportion of SNP with zero effects (*π*) and assuming a common variance for all fitted SNP [11]. A scaled inverse chi-square distribution was used as the prior for the common variance with degrees of freedom υ_*a *_= 4.2 and scale parameter , where  is the additive genetic variance and *p*_*k *_is the allele frequency at locus *k*.

For traits which had early and late records, single trait and bivariate (indicated by adding b to the method's abbreviation) analyses were used for the PBLUP and GBLUP methods. Bivariate analyses were not possible for the Bayesian model averaging methods with the available software. The fixed effect of hatch within generation was fitted in the model for PBLUP and GBLUP. For the Bayesian model averaging methods, data were pre-corrected for fixed effects solutions obtained from PBLUP to enable use of family means to include phenotypes from non-genotyped progeny of genotyped animals (see [10] for details). For the Bayesian model averaging methods, the chain length was 160,000 iterations, with the first 50,000 excluded as the burn-in period. Analyses were performed using the software packages ASREML [12] and GenSel [13].

The accuracy of EBV was evaluated in the validation datasets as the correlation of EBV with hatch corrected phenotype, divided by the square root of heritability, as estimated in single trait PBLUP using all available data. In order to evaluate which factors influence the accuracy of GEBV, the following linear model was fitted to the accuracy obtained from method *i *in generation *j *for trait *k*;

where *b*_*1 *_through *b*_*7 *_are regression coefficients, *h*^*2*^_*k *_is the estimate of heritability for trait *k*, and *π*_*k *_is the estimate of the proportion of SNP with zero effect, as obtained from the BayesCπ method using all data. Accuracy was also evaluated using the above model equation but excluding all terms involving *π *(which is equivalent to fitting average *π*), because in most of the methods *π *is not estimated. The effect of differences between early versus late traits in terms of the number of phenotypes used for training (Table 1) was also evaluated by adding "early versus late" as an effect in the model but this was not found to be significant (p > 0.05) and those results are not reported.

## Results and discussion

### Accuracy in progeny

For all the methods, accuracy varied substantially between traits (Additional file 1, Figure S1), which may be due to differences in heritability, number of records and genetic architecture of traits. Variability in accuracy for the same trait was larger between generations than between methods. This is at least partially explained by the relatively small size of the validation data sets (on average 306 birds/generation), resulting in large sampling errors of the correlation coefficient. Changes in allele frequencies between generations due to drift and selection may have also contributed to variability in accuracy. The observation that differences between methods were small compared to their standard errors was also reported by Luan et al. [14] and Hayes et al. [2].

#### Effect of training size

**Figure 1:**
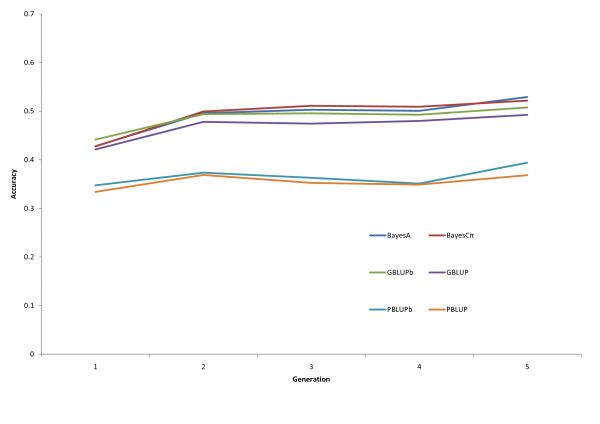
**Accuracy of EBV in progeny from training on accumulated information over generations using pedigree- (PBLUP) or marker-based (GBLUP, BayesA, BayesCπ) methods**. PBLUBb and GBLUPb indicate bivariate analyses; all other methods were single trait

From generation 1 to 5, the number of observations available for training increased about five fold (Table 1). Figure 1 presents the accuracies averaged across traits for the prediction of breeding values in the progeny generation, as information accumulates over generations. For all the methods, accuracy tended to increase with additional information but increases were small after generation 2. The increase was greater for marker-based methods than for PBLUP. According to [2], for a given effective population size, the accuracy of GEBV depends on four parameters: level of LD between markers and QTL, size of the training data set, heritability, and distribution of QTL effects. An increase in accuracy is expected with an increase in size of the training population according to results of simulation studies for both Bayesian model averaging [1] and GBLUP methods [15]. For example, in [1], an increase in training size from 500 to 1,000 individuals led to an increase in accuracy from 0.708 to 0.787 for BayesB and from 0.579 to 0.659 for GBLUP. An increase in accuracy with training data size was also confirmed in real data in several studies [8,11,16]. However, using Australian Holstein-Friesian data, Moser et al. [17] reported that larger training data sets did not result in significant gains in accuracy for GEBV derived using Partial Least Squares Regression. Whereas the studies by Meuwissen [1] and Goddard [15] did not include selection, Muir [7] found that with selection on GEBV, the accuracy of PBLUP could actually exceed accuracy of GEBV over time as the favorable LD erodes with selection on GEBV. However, Sonesson and Meuwissen [5] pointed out that this may not be the case if retraining is done every generation. In our study, selection was on EBV from PBLUP up to generation 3 and on a combination of GEBV and phenotype afterwards, and the advantage of marker-based methods over PBLUP, in terms of accuracy, seemed to slightly increase over generations with increases in the size of the training data.

#### Effect of π and heritability

**Figure 2:**
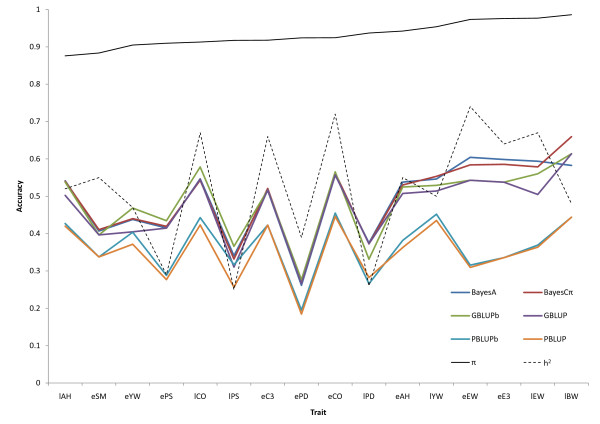
**Average accuracy across generations of EBV in progeny from training on accumulated information using pedigree (PBLUP) and marker based (GBLUP, BayesA, BayesCπ) models**. PBLUBb and GBLUPb indicate bivariate analyses; all other methods were single trait; traits are ranked by the estimate of *π *from model BayesCπ

When all available data were used for training, posterior means for *π *obtained with the BayesCπ method ranged from 0.88 to 0.99, suggesting that from 1 to 12% of the SNP had non-zero effects. Habier et al. [11] showed by simulation that trends in *π *estimates obtained by the BayesCπ method agreed well with the trends in true number of QTL. These estimates were used as the assumed *π *in further analyses. Marker-based methods resulted in greater EBV accuracy than pedigree-based methods for all traits. However, the superiority of the marker-based methods was more pronounced for traits with higher estimates of π, presumably determined by fewer QTL (Figure 2). The difference between pedigree and marker-based methods also increased with heritability, however this trend was less clear than for π (Figure 2).

**Figure 3:**
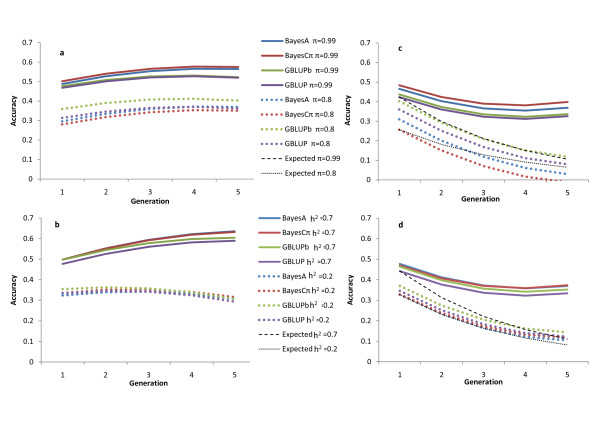
**Least square means for accuracy of marker-based methods over time when predicting progeny using accumulating data (a, b) or when predicting future generations when training on data prior to Generation 1 (c, d)**. In a and c: comparison of accuracy for traits with different estimates of *π *for heritability of 0.5; in b and d: comparison for traits with different heritabilities, ignoring *π*; broken lines are expected declines in accuracy based on declines in genetic relationships

In order to jointly investigate the impact of π and heritability on the accuracy of marker-based models, least square means from the linear regression model fitted to accuracies were obtained. Accuracies from pedigree-based models were omitted in this analysis because they are not expected to depend on π, in the observed *π *range. Heritability (p = 0.028), *π *(p = 0.025), *h*^*2*^_k_*generation (p = 0.002) and generation^2 ^(p = 0.033) had an effect on accuracy at the 0.1 significance level. Results in Figure 3 show that accuracy increased more with the size of the training data for traits with a low estimate of *π *(interpreted as determination by many loci) (Figure 3a) and high heritability (Figure 3b). We observed a higher accuracy for traits with high *π *with all the GEBV estimation methods. Differences between methods were small but the ranking of methods depended on *π *and heritability; with high *π *or high heritability, methods that weighted markers differentially (BayesA and BayesCπ) had the highest accuracy, while GBLUP had the highest accuracy for traits with low *π *or low heritability. Although these differences were not significant, these trends agree with the literature [11,18].

### Persistency of accuracy across generations

#### Method of estimation

**Figure 4:**
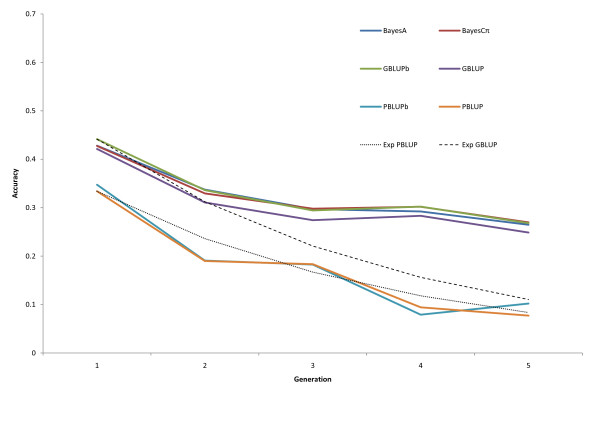
**Accuracy of EBV from training on generation 1 in subsequent generations using pedigree- (PBLUP) and marker-based (GBLUP, BayesA, BayesCπ) models, compared to the expected decline of accuracy based only on the decay of relationships (Exp PBLUP, Exp GBLUP)**.

Figure 4 shows the accuracies of pedigree and genomic EBV per generation, averaged over the 16 traits, when training only on data up to generation one. This rate of decline was observed for pedigree-based EBV but the accuracies of GEBV were more persistent. With the pedigree-based analysis, when validation and training were more than three generations apart, the predictive ability for some traits was practically zero (Additional file 2, Figure S2). For marker-based methods, the accuracy also dropped over time but substantial accuracy was retained even after five generations. The drop in accuracy was greatest in the first generation, likely because of loss of information through pedigree relationships. The highest drop in accuracy in the first generation after the phenotyping was stopped was also observed in simulation studies [4,5]. We did not observe large differences between GBLUP and Bayesian model averaging methods in persistency of accuracy. This is in agreement with results from [8], except for milk yield, where BayesB was more persistent. Where available, EBV from bivariate analyses were slightly more predictive than EBV from single-trait analyses (Figure 2).

#### Effect of π and heritability

At the 0.1 significance level, *h*^*2 *^(p = 0.063), generation (p = 0.002), generation^2 ^(p = 0.0001), π*generation (p = 0.054), and *h*^2^*generation (p = 0.092) had an effect on accuracy. Least square means from the linear regression model (Figure 3C) suggest higher persistency of accuracy over time for traits with high estimates of *π *(egg weight, body weight), indicating that the GEBV for these traits capture more information through LD or linkage with QTL, compared to traits which seem to be more polygenic and where markers explain mostly pedigree relationships. Traits with a low heritability showed less persistent accuracy.

With five generations separating training and validation, accuracy in the last generation in Additional file 2, Figure S2 should be a good estimate of accuracy due to LD. Goddard [15] showed that accuracy due to LD from GBLUP depends on the number of independent chromosomal segments. Daetwyler et al. [18] have shown that accuracy due to LD is expected to depend on the smaller of the number of QTL and the number of independent chromosomal segments for Bayesian model averaging methods. On the other hand, they reasoned that accuracy for GBLUP does not depend on the number of QTL [18]. In our study, the average accuracy for the four traits with the highest *π *estimates was 0.52, 0.48 and 0.43 for BayesCπ, BayesA and GBLUPb, and 0.23, 0.23 and 0.24 for the four traits with the lowest *π*. This confirms the theoretical prediction that Bayesian model averaging methods have a higher accuracy for traits that are determined by a small number of genes compared to traits determined by many genes, as classified according to the *π *estimate. However, our results show that accuracy of GBLUP changed with *π*, in contrast to what was predicted by [18] but this may be caused by other factors that can contribute to the differences in accuracies between traits, such as the distribution of QTL effects and non-additive or more complex types of genetic determination. On average, accuracy due to LD (retained after five generations) was similar for all marker-based methods, as was also the rate of decline in accuracy. Habier et al. [8] reported similar results for somatic cell score and fat yield, however, they found that for other traits, accuracy due to LD of BayesB was higher than for GBLUP, especially for larger training size. Based on simulation results [19], accuracy and its persistency are expected to increase with increasing marker density for the BayesB method.

## Conclusions

Using real data on egg production and quality traits in layers, this study confirms that the accuracy of EBV based on dense marker data is on average higher than that based on pedigree. An increase in size of the training data improved the accuracy of all methods but not to the degree expected based on theory, possibly as a result of the population being under selection. Marker-based methods had greater advantage over PBLUP, in terms of accuracy, for traits with a high estimate of *π*, which implies a small number of QTL with large effects. There were no significant differences between BayesA, BayesCπ and GBLUP methods in average accuracy or persistency of accuracy across traits. The accuracy of the marker-based EBV was more persistent than that of pedigree-based EBV over generations, which indicates that markers capture information on QTL through LD and/or linkage. However, the initial drop of accuracy due to loss of pedigree relationships limits the value of genomic predictions further than one generation ahead. When training was on data from generation 0, accuracy in generation 1 was 0.43 and dropped to 0.33 in generation 2. However, when data from generations 0 and 1 were used for retraining, accuracy in generation 2 increased to 0.49. Retraining every generation is recommended for genomic selection in closed breeding populations.

## Competing interests

The authors declare that they have no competing interests.

## Authors' contributions

All authors conceived the study, contributed to methods and to writing the paper and also read and approved the final manuscript. AW undertook the analysis and wrote the first draft. Data were prepared by JA, PS, JF and NPO. JCMD provided overall oversight of the project.

## Supplementary Material

Additional file 1 **Figure S1 - Accuracy of prediction in the progeny generation with accumulating data**. A: PBLUPb; B: BayesA; C: GBLUPb; D: BayesCπ Click here for file

Additional file 2 **Figure S2 - Accuracy of prediction in subsequent generations when training on data prior to Generation 1**. A: PBLUPb; B: BayesA; C: GBLUPb; D: BayesCπ Click here for file
