# Supplementary figures and images for: Persistence of accuracy of genomic estimated breeding values over generations in layer chickens
Source: Genet Sel Evol. 2011 Jun 21;43(1):23. doi: 10.1186/1297-9686-43-23 (PMC3144444; doi:10.1186/1297-9686-43-23)

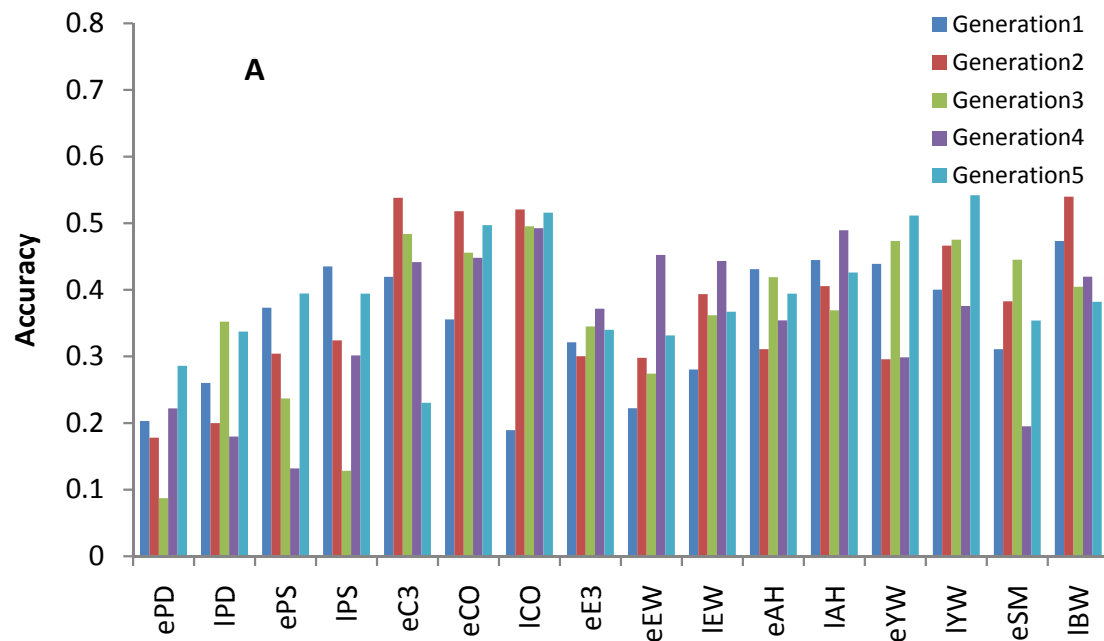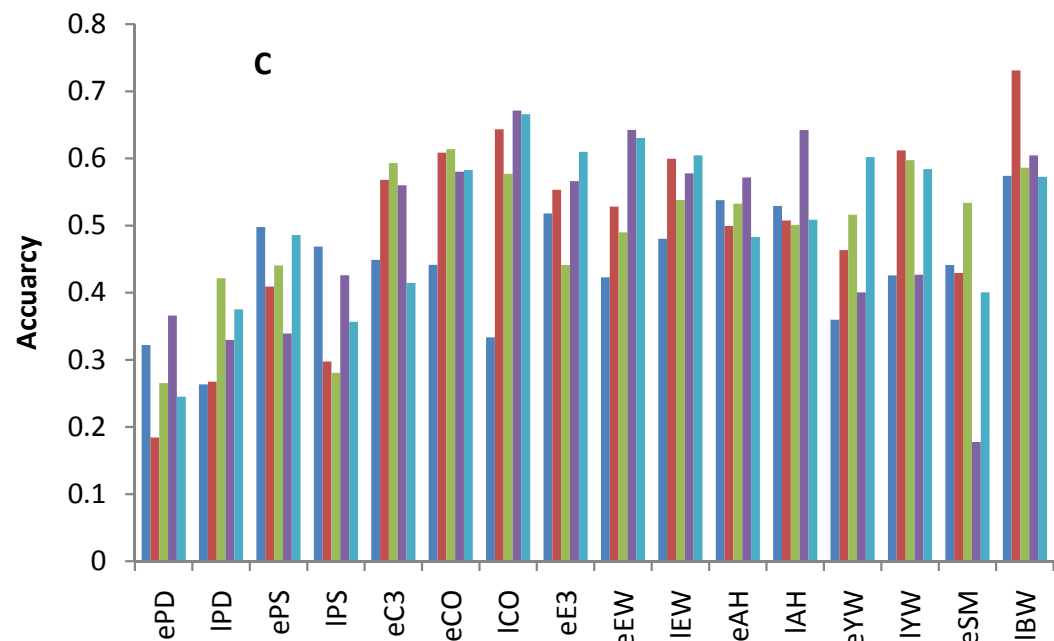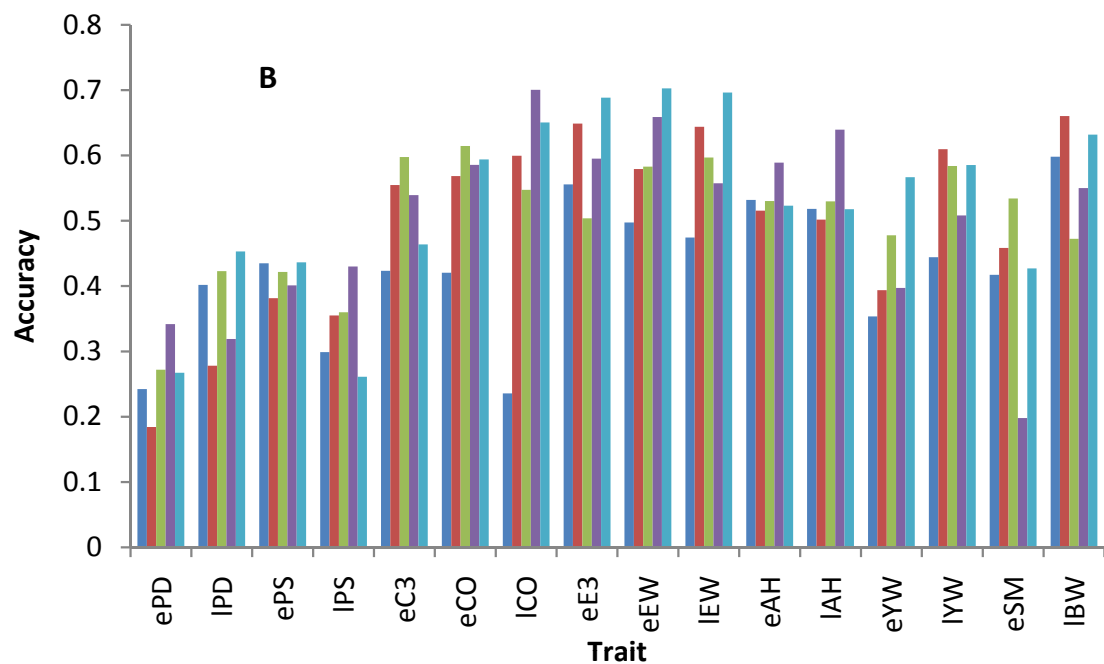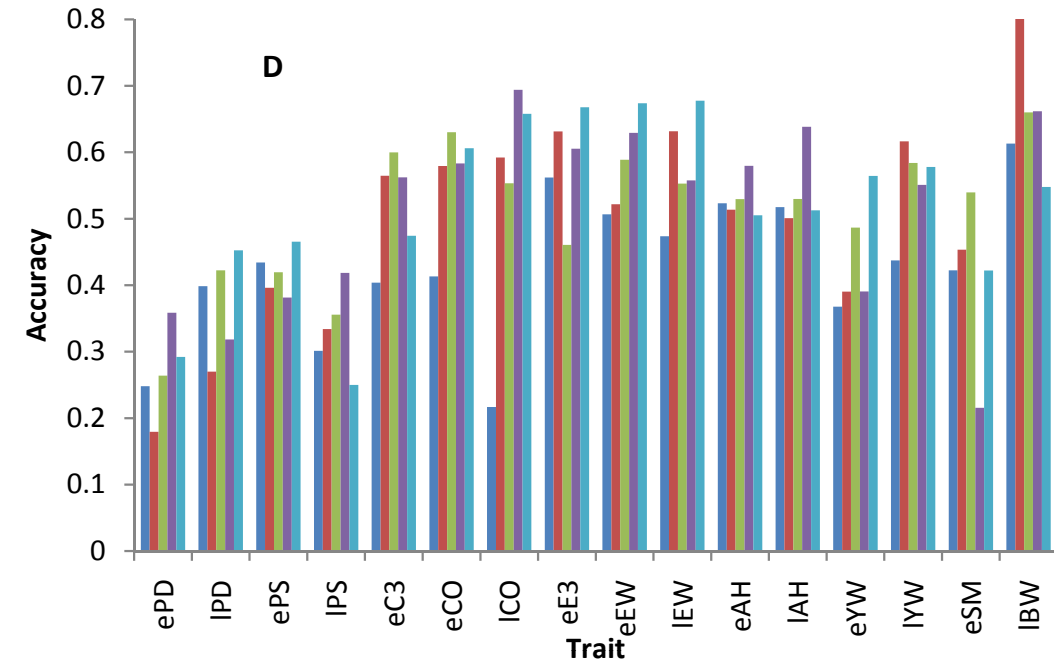

Supplement: Additional file 1 — Figure S1 - Accuracy of prediction in the progeny generation with accumulating data. A: PBLUPb; B: BayesA; C: GBLUPb; D: BayesCπ [file 1297-9686-43-23-S1.PDF]

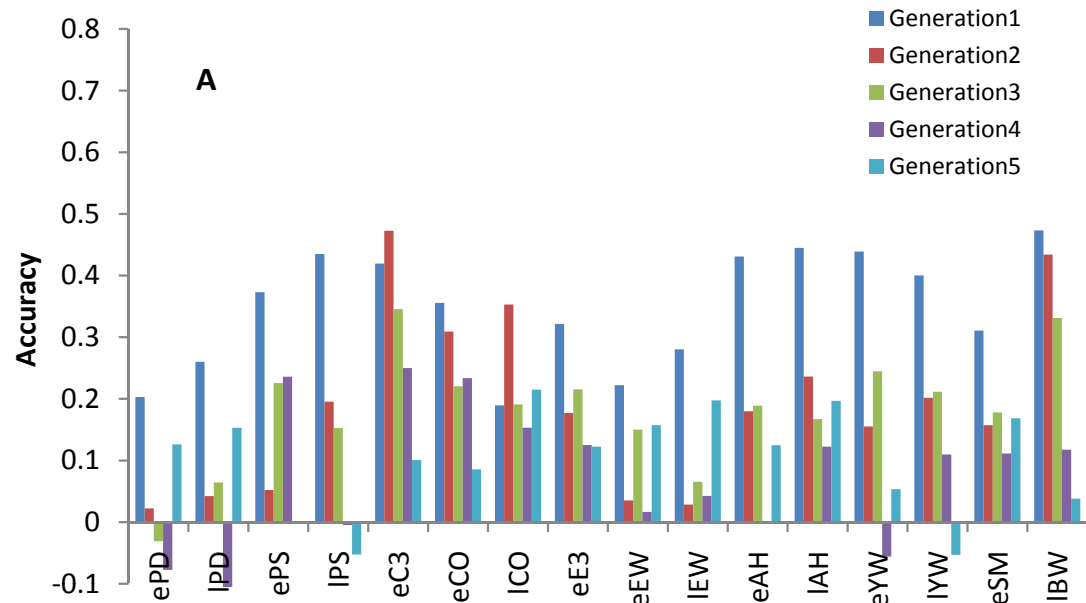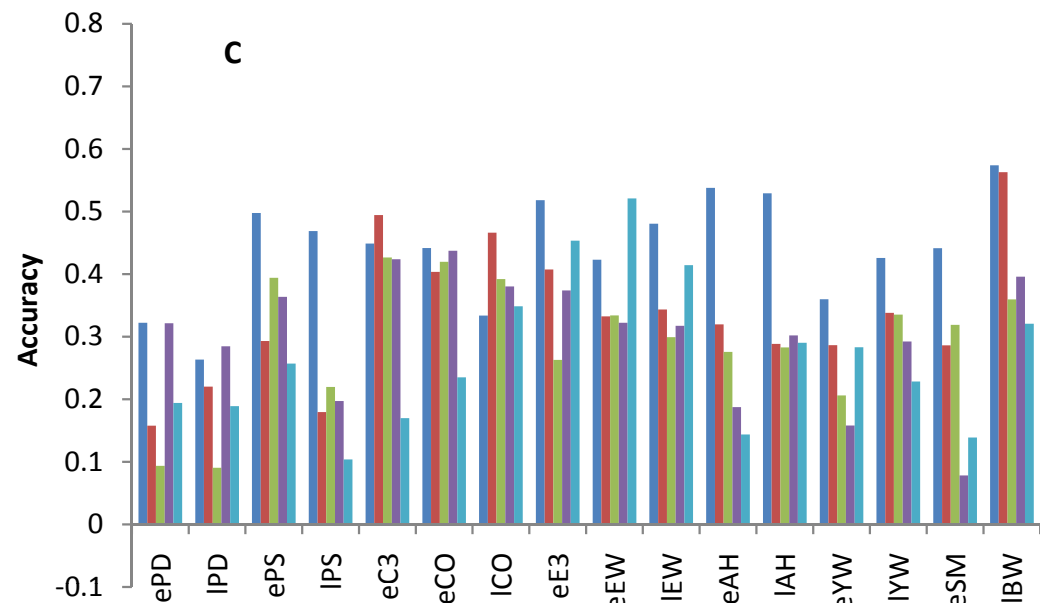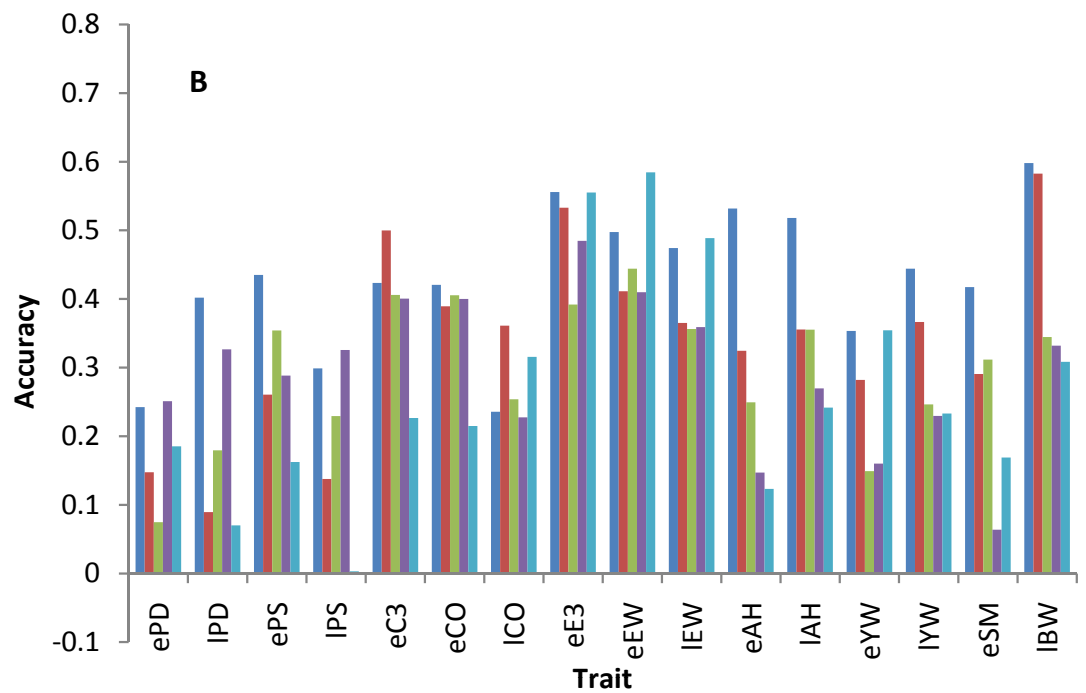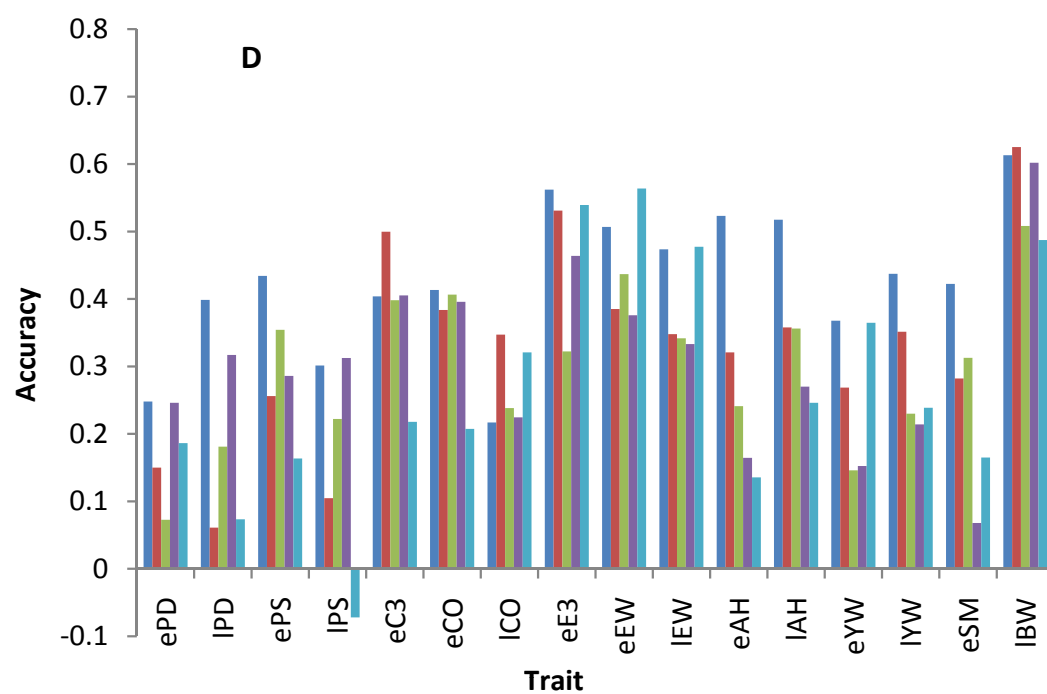

Supplement: Additional file 2 — Figure S2 - Accuracy of prediction in subsequent generations when training on data prior to Generation 1. A: PBLUPb; B: BayesA; C: GBLUPb; D: BayesCπ [file 1297-9686-43-23-S2.PDF]
